# Supplementary material for: Sentence processing is modulated by the current linguistic environment and a priori information: An fMRI study
Source: Brain Behav. 2019 Jun 14;9(7):e01308. doi: 10.1002/brb3.1308 (PMC6625468; doi:10.1002/brb3.1308)
Supplement: Supplementary file 1 [file BRB3-9-e01308-s001.docx]

**Supplementary Table 1**. Whole-brain effects of the parametric modulations of verb-based syntactic surprisal using the individual verb bias values per subject (N=17). Listed are local maxima more than 20mm apart. All clusters at a voxel-level threshold of p<.005, k=100 are reported (note the less conservative threshold as this contrast is on less participants and serves mainly to illustrate the pattern overlap with the surprisal analysis using group-average surprisal values in the main text), those that reach cluster-level FWE correction or small volume correction at p<.05 (or p<.025 for the 2 planned comparisons) are marked by ‡.

| **Anatomical label** | **BA** | **Global and local maxima** | | | **cluster size k** | **cluster-level pFWE<.05** | **Z** |
| --- | --- | --- | --- | --- | --- | --- | --- |
|  |  | **x** | **y** | **z** |  |  |  |
| **Parametric modulation verb-based syntactic surprisal** | | | | | | | |
| x |  |  |  |  |  |  |  |
| **Parametric modulation verb-based syntactic surprisal; DO structure** | | | | | | | |
| Right putamen |  | 32 | -2 | 12 | 703 | 0.009 ‡ | 4.59 |
| Right rolandic operculum |  | 40 | -20 | 22 |  |  | 3.66 |
| Right inferior frontal gyrus (pars orbitalis) | 47 | 40 | 38 | -4 | 272 | 0.281 | 4.37 |
| Left thalamus |  | -26 | -24 | 12 | 227 | 0.406 | 4.26 |
| Left pallidum |  | -16 | -6 | 10 |  |  | 3.80 |
| Left inferior frontal gyrus (pars orbitalis) | 47 | -42 | 32 | -6 | 246 | 0.348 | 4.22 |
| left fusiform gyrus | 19/37 | -42 | -68 | -10 | 1078 | 0.001 ‡ | 4.06 |
| Left fusiform gyrus | 37 | -40 | -46 | -20 |  |  | 3.80 |
| Left cerebellum (Lobule V) |  | -18 | -50 | -16 |  |  | 3.72 |
| Right middle occipital gyrus | 19 | 46 | -82 | 12 | 137 | 0.766 | 4.04 |
| Right inferior/middle temporal gyrus | 37 | 48 | -66 | 0 |  |  | 3.11 |
| Left middle temporal gyrus | 21/39 | -48 | -58 | 24 | 176 | 0.598 | 4.04 |
| Left middle temporal gyrus | 39 | -48 | -62 | 26 |  | pSVC=.049 | 3.5 |
| (IPS) | 40 | 26 | -46 | 36 | 172 | 0.615 | 3.93 |
| Right superior occipital gyrus | 19 | 24 | -66 | 34 |  |  | 3.06 |
| Left precentral gyrus | 6 | -60 | 2 | 34 | 106 | 0.886 | 3.90 |
| Left inferior frontal gyrus (triangularis) | 45 | -56 | 20 | 24 |  | pSVC=.27 | 2.86 |
| Left middle temporal gyrus | 21/22 | -56 | -18 | -4 | 183 | 0.569 (pSVC=.019) ‡ | 3.79 |
| Right superior temporal gyrus | 22 | 66 | -20 | 8 | 153 | 0.697 | 3.61 |
| Right superior temporal gyrus | 22/42 | 56 | -38 | 12 |  |  | 3.48 |
| Right cerebellum (Lobule I IV) | 30 | 16 | -32 | -22 | 140 | 0.753 | 3.40 |
| Right cerebellum |  | -2 | -32 | -8 |  |  | 2.70 |
| Left inferior parietal lobule | 7 | -30 | -72 | 46 | 119 | 0.839 | 3.35 |
| Right middle occipital gyrus | 19 | 38 | -84 | 32 | 106 | 0.886 | 3.27 |
| **Parametric modulation verb-based syntactic surprisal; PO structure** | | | | | | | |
| x |  |  |  |  |  |  |  |
| **Parametric modulation verb-based syntactic surprisal by ‘Current Structure Statistics‘ by type of structure** | | | | | | | |
| x |  |  |  |  |  |  |  |

**
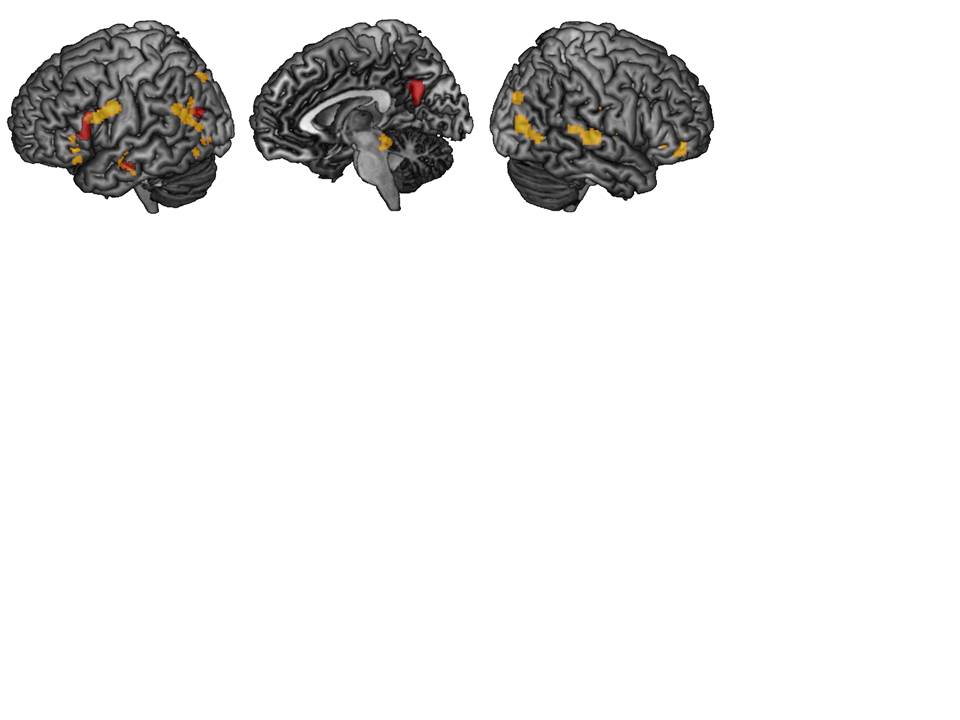
**

**Supplementary Figure 1.** Whole-brain effects of the parametric modulations of verb-based syntactic surprisal using the individual verb bias values per subject (N=17) in yellow (voxel-level p=.005, k=100) and the parametric modulations of verb-based syntactic surprisal using the group average values, as reported in the main text, in red (voxel-level p=.001, k=25).
